# Supplementary material for: A scalable and cGMP-compatible autologous organotypic cell therapy for Dystrophic Epidermolysis Bullosa
Source: Nat Commun. 2024 Jul 11;15:5834. doi: 10.1038/s41467-024-49400-z (PMC11239819; doi:10.1038/s41467-024-49400-z)
Supplement: Supplementary file 6 — Supplementary Data 3 [file 41467_2024_49400_MOESM6_ESM.zip › Supplementary Data 3/57_offtarget_sites/CO1-173_2MB/patient3_2.chr1_17258328_17258350.2MB.pdf]

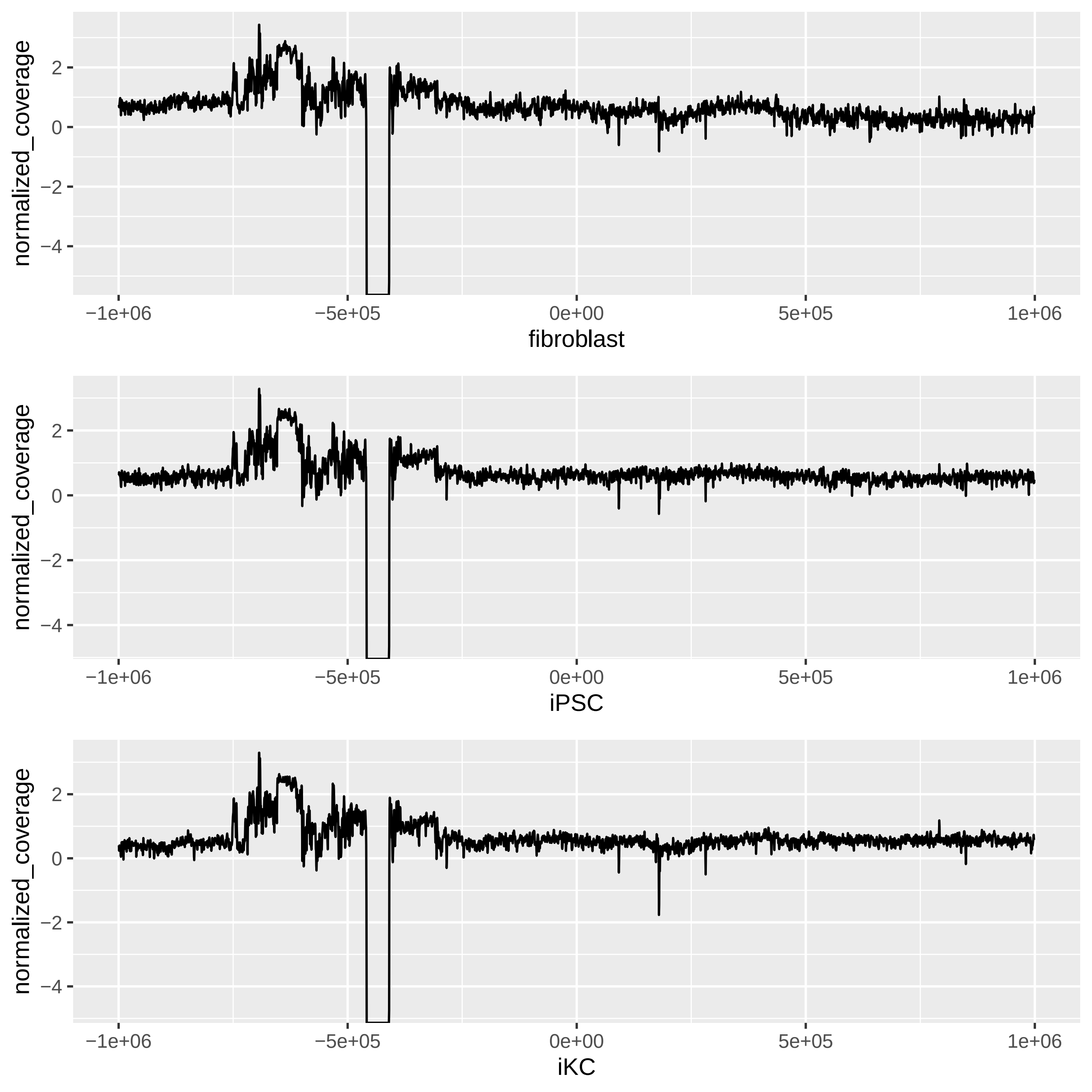
normalized_coverage
2
0
−2
−4
−1e+06
−5e+05
0e+00
5e+05
1e+06
fibroblast
normalized_coverage
2
0
−2
−4
−1e+06
−5e+05
0e+00
5e+05
1e+06
iPSC
normalized_coverage
2
0
−2
−4
−1e+06
−5e+05
0e+00
5e+05
1e+06
iKC
